# Elevated CO_2_ and food ration affect growth but not the size-based hierarchy of a reef fish

**DOI:** 10.1038/s41598-019-56002-z

**Published:** 2019-12-23

**Authors:** Shannon J. McMahon, Philip L. Munday, Marian Y. L. Wong, Jennifer M. Donelson

**Affiliations:** 1 ARC Centre of Excellence for Coral Reef Studies, James Cook University, Townsville, QLD 4811 Australia; 2 Centre for Sustainable Ecosystem Solutions, School of Biological Sciences, University of Wollongong, Wollongong, NSW 2522 Australia

**Keywords:** Climate-change ecology, Climate-change impacts

## Abstract

Under projected levels of ocean acidification, shifts in energetic demands and food availability could interact to effect the growth and development of marine organisms. Changes to individual growth rates could then flow on to influence emergent properties of social groups, particularly in species that form size-based hierarchies. To test the potential interactive effects of (1) food availability, (2) elevated CO_2_ during juvenile development, and (3) parental experience of elevated CO_2_ on the growth, condition and size-based hierarchy of juvenile fish, we reared orange clownfish (*Amphiprion percula*) for 50 days post-hatching in a fully orthogonal design. Development in elevated CO_2_ reduced standard length and weight of juveniles, by 9% and 11% respectively, compared to ambient. Development under low food availability reduced length and weight of juveniles by 7% and 15% respectively, compared to high food. Parental exposure to elevated CO_2_ restored the length of juveniles to that of controls, but it did not restore weight, resulting in juveniles from elevated CO_2_ parents exhibiting 33% lower body condition when reared in elevated CO_2_. The body size ratios (relative size of a fish from the rank above) within juvenile groups were not affected by any treatment, suggesting relative robustness of group-level structure despite alterations in individual size and condition. This study demonstrates that both food availability and elevated CO_2_ can influence the physical attributes of juvenile reef fish, but these changes may not disrupt the emergent group structure of this social species, at least amongst juveniles.

## Introduction

The availability of sufficient energetic resources to meet biological energy demands is essential to individual fitness. Energy intake is firstly used to maintain essential cellular processes, with any surplus energy divided among non-essential processes (e.g. growth, reproduction and muscle development) and building energy reserves^1–3^. In natural environments, energetic resources are finite and consequently the availability of energy to an individual can be insufficient to meet all additional energetic demands above basic cell processes^4–6^. Consequently, trade-offs occur within non-essential processes, such that some are provided more energy than others^3,7,8^. Where exactly energetic resources are partitioned will have implications for the fitness of the individual^9,10^. This is especially true during early life stages where juveniles have high energetic demands to sustain their growth and development^10,11^. Thus, environmental changes that influence either the supply of energy, or the costs of cellular processes, have the potential to alter energy budgets, and consequently, individual performance. Changes to individual performance can then potentially flow on to affect emergent traits of the population^12^, which may be especially significant for social species where body size is a key factor in social interactions.

One environmental challenge that can alter both the resources available to marine organisms, as well as the cellular costs of basic maintenance, is the rising atmospheric CO_2_ level and subsequent increase in ocean pCO_2_^13–15^. Higher seawater pCO_2_ increases the energetic cost of acid-base regulation to maintain a favourable internal pH^16–18^. Increased energetic demands to maintain the preferred pH for cellular processes may divert energy from other processes. Indeed, recent research has shown that elevated CO_2_ exposure results in reduced growth and survival during the early life history of some fishes^19–23^, possibly due to increased energetic costs of cellular processes. In addition, climate change is expected to reduce primary production, which will result in lower resource availability for fish through trophic effects^24–26^. Therefore, compensatory foraging may not be a viable method to meet the energy requirements for growth and other costly processes in the future, in addition to the increase costs of maintaining homeostasis in high CO_2_ conditions^27^.

For animals that live in social groups, their access to resources and patterns of growth are also dependent on other individuals in the group. One organisational structure that social fishes employ is a size-based social hierarchy, with individuals in rank order by their relative size within a group^28–30^. This social structure is thought to mediate social competition and reduce conflict within the group while individuals queue to inherit limiting breeding positions^31,32^. Size-based social hierarchies are typically behaviourally regulated, with subordinate individuals limiting their food intake and hence regulating their growth in order to maintain the appropriate body size ratio in relation to the next fish in the social hierarchy^33^. If individuals approach a size too close to the rank above they can cause conflict, which may result in their eviction form the colony^28,33^. Despite the fact that elevated ocean CO_2_ can affect numerous behavioural responses in individual fish, including anti-predator response^34–37^, olfactory, auditory and visual preferences^38–41^, activity levels^42–44^ and learning^45,46^, very little is known about the potential impacts that altered individual responses could subsequently have at the group-level. While there have been a few studies investigating the effects of CO_2_ on social behaviour^47–49^, none to date have investigated how size-based hierarchies may be affected. Therefore, the possibility that behaviourally and growth regulated size hierarchies may also be altered under future CO_2_ conditions, in response to altered individual attributes, remains untested.

Changes to ocean conditions, including rising pCO_2_, will not occur within a single generation for most marine organisms. Thus, it is essential to examine the effect that environmental change in one generation may have on the next^50^. Parents have the capacity to alter their offspring phenotype in relation to the conditions they have experienced. This may occur in an adaptive way, where offspring are better suited to environmental conditions^51–53^, or alternatively, stressful parental conditions can negatively impact offspring performance^52,54^. Parental effects come about by a range of different mechanisms, such as differential nutritional provisioning, transfer of hormones and proteins, epigenetic changes and even behavioural learning^55–57^. In relation to elevated CO_2_, parental exposure has been shown to partially or fully mitigate negative effects of elevated CO_2_ on offspring traits such as escape performance^58^, survival and growth^59–61^. In contrast, there was little improvement in behavioural responses to chemical cues or behavioural lateralization when juvenile fish and their parents were exposed to elevated CO_2_^36,37^. The diversity of responses previously observed when both parents and their offspring experience elevated CO_2_ conditions highlights the need for further research to understand how parental effects may influence offspring performance in future projected ocean conditions, especially when food resources are limited.

In this study, we investigated the interacting effects that elevated CO_2_ and food supply have on growth of a juvenile reef fish and if this has flow-on effects to their social organisation. Specifically, we tested the effects of elevated CO_2_ and limited food supply on growth (standard length and weight) and body condition (Fulton’s K) in the orange clownfish, *Amphiprion percula*, as a model marine fish species that forms size-hierarchies^28,62^. We aimed to determine how parental exposure to elevated CO_2_, juvenile development in elevated CO_2_, and difference in food availability, influence the growth and body condition of juveniles, and whether the combination of these factors have an interactive effect on juvenile fish that would not be predictable when testing each in isolation. Furthermore, by comparing sized-based hierarchies among treatments we investigated how the effects of cross-generational elevated CO_2_ or food availability (or their interaction) on individuals could flow through to affect the emergent size-based structure of fish social groups.

## Results

**Figure 1:**
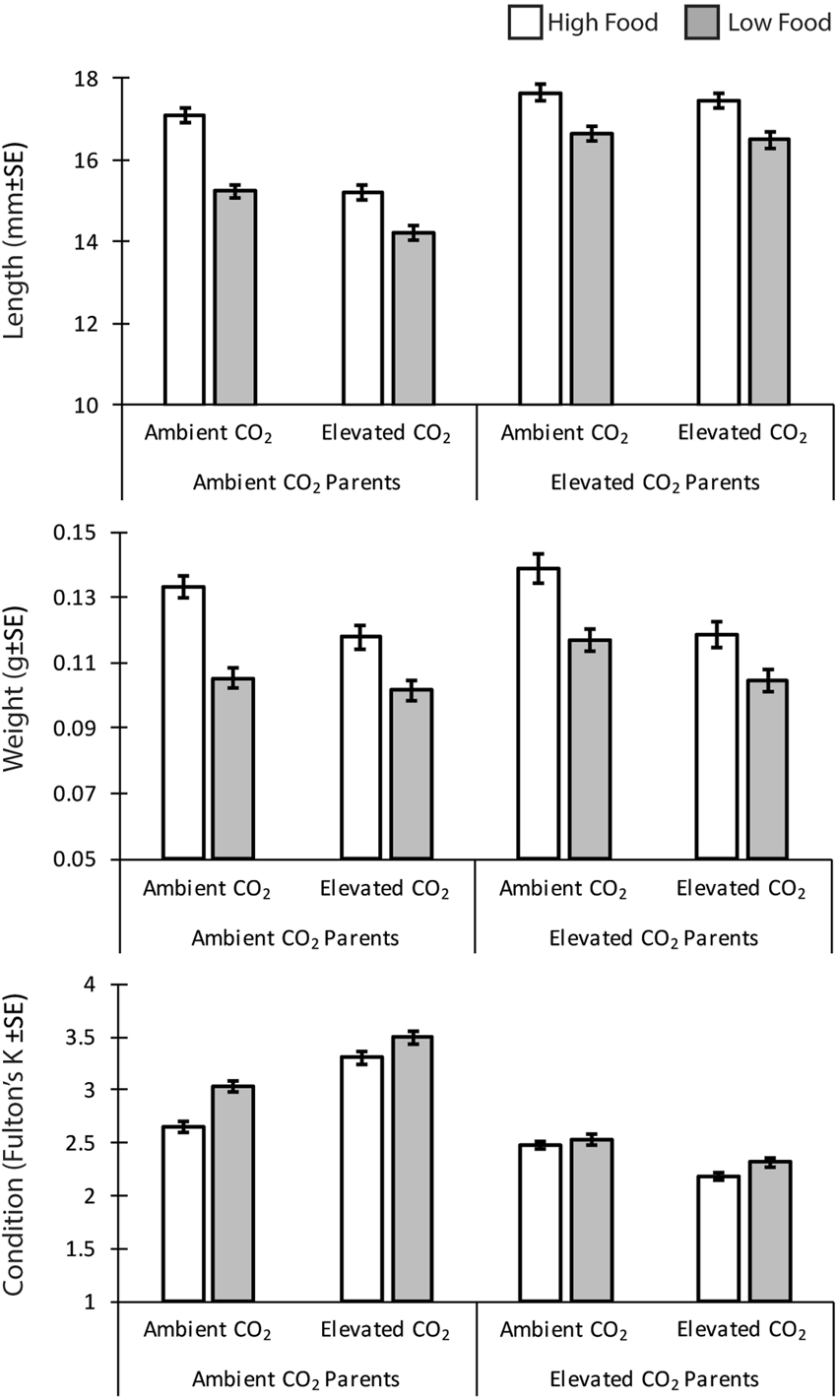
Average (±SE) of standard length, weight, and condition of juvenile *A. percula*. Juveniles were reared in either high (white bars) or low food (grey bars) treatment cross factored with ambient (489 µatm) or elevated CO_2_ (1022 µatm). Juveniles were from parents exposed to either ambient (489 µatm) or elevated CO_2_ (1032 µatm).

Juvenile fish reared on the low food ration were significantly shorter (F_1,57_ = 28.11, P < 0.001), lighter (F_1,57_ = 21.19, P < 0.001) and in poorer condition (Fulton’s K index; F_1,57_ = 10.62, P = 0.002) at 50 dph than those provided the high food ration, regardless of parental or juvenile CO_2_ treatment (Fig. 1; Tables S1–S3). Specifically, juveniles were on average 7% shorter and weighed 15% less than their high food counterparts regardless of parental or juvenile CO_2_ treatment.

The length of juveniles at 50 dph was also influenced by a combination of parental and juvenile CO_2_ treatments (F_1,57_ = 8.03, P = 0.006). Generally, juveniles from elevated CO_2_ parents were 10% larger than juveniles from control CO_2_ parents. Juveniles from control CO_2_ treatment parents that developed in the elevated CO_2_ conditions were 9% shorter compared to siblings that developed at control CO_2_ (Table S6). However, if parental fish were maintained in elevated CO_2_ conditions there was <1% difference in the size of juveniles in the elevated CO_2_ treatment compared with the ambient current-day control (Fig. 1) (Table S6).

The weight of juveniles was significantly affected by juvenile CO_2_ conditions, with fish that developed in elevated CO_2_ conditions weighing 11% less than those reared in current-day control conditions (Fig. 1; F_1,57_ = 10.88, P = 0.002). This pattern was consistent across parental CO_2_ treatment and there was no evidence that parental treatment affected the weight of juveniles (F_1,7_ = 0.16, P = 0.701).

These differences in length and weight resulted in an interactive effect of parental and juvenile CO_2_ treatments on Fulton’s K condition (F_1,57_ = 50.27, P < 0.001). Specifically, offspring from control CO_2_ parents were in significantly better physical condition (33%) when they developed in elevated CO_2_ (Table S7). While offspring from elevated CO_2_ parents were found to be in slightly poorer (12%) condition when grown in elevated CO_2_ conditions, compared to control juvenile CO_2_ conditions (Table S7).

Individual length, weight and Fulton’s K condition all declined significantly as the size rank of the juvenile within the tank increased (SL: F_9,576_ = 439.15, P = < 0.001, W: F_9,576_ = 109.48, P < 0.001 and FK: F_9,576_ = 50.27, P < 0.001), as would be expected with a size based hierarchal structure. There was no evidence that the size rank of an individual was differentially affected by any of the treatments, with no interactions found between size rank and any combination of the treatments (Table S3).

**Figure 2:**
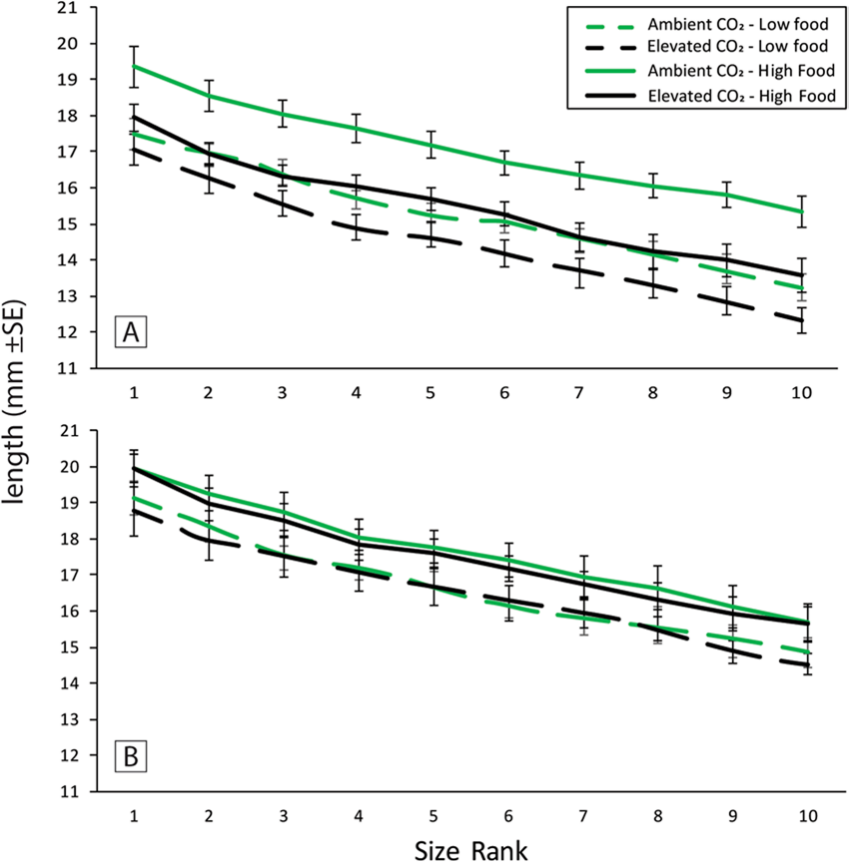
Average length (±SE) of each rank within the size hierarchy of *A. percula*. Juveniles from either ambient CO_2_ (489 µatm) treated parents (**A**) or elevated CO_2_ (1032 µatm) treated parents (**B**). Juveniles were in a cross-factored treatment design with ambient (489 µatm) or elevated CO_2_ (1022 µatm), and low or high food treatments.

To further explore the relationship of growth between individuals and the maintenance of the size hierarchy, the frequency of body size ratios was explored. Generally, body size ratios (SL of rank N/SL of rank N + 1) were most frequently in the range of 0.925 to 0.999 relative to the individual ranked immediately above (Fig. S1). Body size ratios were not significantly affected by juvenile food treatment (F_1,448_ = 0.031, P = 0.850), parental CO_2_ treatment (F_1,448_ = 0.033, P = 0.855), or juvenile CO_2_ treatment (F_1,448_ = 1.271, P = 0.260). There were also no significant interactions (Table S4). The length of rank 1 individuals differed depending on food treatments (F_1,57_ = 17.59, P = < 0.001), juvenile CO_2_ (F_1,57_ = 6.04, P = 0.017) and parental CO_2_ exposure (F_1,7_ = 6.36, P = 0.04) (Table S5). This resulted in shifts of the overall body size of individuals within groups but did not affect body size ratios in any of the treatments (Fig. 2).

## Discussion

The growth and physical condition of juvenile fish is critical to their performance and survival^63–66^, yet how these traits are affected by the environmental conditions experienced during early life, and the conditions experienced by their parents, is still poorly understood. This study found that the CO_2_ conditions experienced by the parents, as well as the food ration and CO_2_ levels experienced by juveniles, all influenced the growth and body condition of juvenile clownfish. As expected, food ration had a clear effect on growth and physical condition of juveniles. Fish on the low food ration were shorter and lighter than their counterparts on high food rations, and this effect was consistent among juvenile and parental CO_2_ treatments. However, when parents were exposed to elevated CO_2_ conditions their offspring exhibited enhanced linear growth, especially when they developed in elevated CO_2_ conditions, compared to offspring from control CO_2_ parents. Enhanced linear growth may be linked to the need to maintain the size-based hierarchy within this species^28,33^. By contrast, parental exposure to elevated CO_2_ did not affect juvenile weight in any treatment. The increase in length but not weight resulted in a lower physical condition (Fulton’s K) in juveniles of elevated CO_2_ parents. These results show that both altered energetic resources and elevated CO_2_ levels projected by the end of this century have the potential to impact the growth and body condition of *A. percula*; however, these changes do not necessarily flow on to affect higher order traits, such as the structure of the social hierarchy.

The food resources available to an individual relative to its basic energetic demands will determine the surplus energy available for other processes including growth^3,8^. In this study, juveniles reared on a low food ration were shorter and lighter in all CO_2_ treatments. Exposure of juveniles to elevated CO_2_ also reduced their length and weight, but only when their parents experienced ambient CO_2_ conditions. Juveniles from elevated CO_2_ parents grew similarly in either ambient or elevated juvenile conditions to offspring from control CO_2_ parents reared at ambient conditions. This suggests that exposure of parents to elevated CO_2_ may have preconditioned offspring with enhanced linear growth capacity under elevated CO_2_ conditions. Beneficial effects of parental CO_2_ exposure did not extend to enhancement of weight and body condition, indicating that parental exposure could only partially compensate for the costs of elevated CO_2_ during juvenile development. While juveniles from elevated CO_2_ parents were able to put more energy into linear growth, they did not increase in weight. Therefore, enhanced linear growth came at the cost of poorer physical condition, with lower mass for a given length in the juveniles produced by elevated CO_2_ parents. This suggests that an energetic trade-off was likely made to favour linear growth over an increase in weight, resulting in lower body condition. Overall the effects of food availability and juvenile CO_2_ exposure resulted in similar magnitudes of effects on growth and condition. For example, half the available food resulted in a 7% change in length, which was the same reduction as observed for fish that experienced elevated juvenile CO_2_ conditions from control parents. Similarly, reduced food produced a 15% reduction in the weight of juveniles compared to 11% reduction with development in elevated CO_2_ regardless of parental conditions. Reduced length and weight is likely to have negative consequences for juveniles in the wild, likley resulting in higher predation^67^  as smaller sized individuals are more likely to be predated upon^68^. Therefore, changes in juvenile size and condition would effect individual survival, which could potentially flow on to effect recruitment at the population level^63,65,66^.

While both elevated CO_2_ during juvenile development and food availability affected growth and condition there was no interaction between these two factors. This additive effect is consistent with a recent study by Gobler and colleagues^27^, who also found that food ration and elevated CO_2_ had additive, but not interactive, effects on growth and survival in two species of coastal fishes. The absence of an interaction between food ration and juvenile CO_2_ treatment suggests that variation in food supply under future conditions will not act synergistically with ocean acidification conditions, and could make predicting future effects to fisheries and fish species easier. However, our results also contrast with some previous research on invertebrates, which has found that the effects of ocean acidification are up to three times stronger under reduced energy supply^69,70^. These dissimilar results suggest that elevated CO_2_ is less physiologically stressful in fish than it is to some invertebrates, possibly because of the efficient acid-base regulatory abilities of most fishes^16–18^, or that our food levels used were still within a range that is not limiting.

The CO_2_ conditions experienced by parents influenced the phenotypic response of their offspring, increasing linear growth but reducing physical condition. In a broad range of taxa, parental exposure to stressful conditions influences offspring phenotype, and in some cases it enhances offspring phenotype under the same stressful conditions^54,71,72^. This cross-generational restoration of growth in elevated CO_2_ has been previously observed in another anemonefish species^59^ and could be associated with changes in gene expression as a result of high CO_2_ exposure of the parents^73^. In this previous study^59^, we do not know how juveniles from elevated CO_2_ parents would perform back in ambient control conditions, but in the current research we found that enhanced linear growth was observed in both juvenile CO_2_ conditions, not only when parental and offspring conditions matched.

While it was clear that food availability altered juvenile growth, and parental and juvenile CO_2_ treatment interacted to influence the length of juveniles, the relative size ratio between ranks was not affected. The largest juvenile in each group was smaller on the low food ration and in the juvenile elevated CO_2_ treatment, but the relative size ratio between all juveniles of sequentially smaller size remained constant. It is important to note that in order to successfully conduct this experiment we had to deviate from a natural hierarchy, by using a larger number of individuals per group that consisted of siblings of the same size and age. While this was done in order to ensure sufficient numbers of fish were present by the end of the experiment, this set up does not replicate the natural structure and functioning of wild anemonefish groups. Even so, these results provide at least an initial insight into how size based social hierarchies amongst juveniles may be affected by elevated CO_2_ and food availability, given that juvenile reef fish can recruit together at the same time^74,75^. In addition, the absence of an effect of food ration or elevated CO_2_ on body size ratios suggests that the processes and pay-offs surrounding social conflict and its resolution within groups in *A. percula* may be robust to shifts in abiotic parameters. If subordinate individuals become too large compared with their immediate dominant they are likely to be evicted from the social group^76^. Therefore, the potential ramifications of not maintaining the correct size difference relative to the immediate dominant could explain why the body length of ranks shifted uniformly under the various treatments^76,77^. Even when food is abundant, individuals in size-based hierarchies can modulate their feeding rate to prevent growing too large compared with their immediate dominant^33^. Our results, wherein the high food ration did not lead to larger size ratios, supports the prevailing notion that subordinates regulate their feeding so as to regulate their growth, and thus their relative length compared with others in the social group^33^.

Emerging research shows differing effects of elevated CO_2_ depending on the level of traits or performance investigated. For example, Goldenberg and colleagues^78^ found that while fish exposed to elevated CO_2_ had impaired visual and olfactory performance effecting the ability to locate prey, the feeding efficiency of these fish in a mesocosm setting was unaffected. The present finding shows a similar mismatch between the social hierarchy robustness compared to the impaired predator avoidance on the basis of olfactory cues for the same fish used in this study^36^. In contrast, short-term exposure to elevated CO_2_ has been found to reduce cohesion in fish shoals^47^ and reduce the familiarity of shoal members to one another^48^. Further research is needed to understand the consistency of CO_2_ effects to various levels of behaviour and social organisation^79^. One possibility for diversity of results, is that the regulatory mechanisms controlling growth rate in size-based hierarchies are different from the effects of elevated CO_2_ on neurological processes associated with other behaviours, such as anti-predator responses, lateralization and learning^14,45,62,73^.

Both the availability of energetic resources and the CO_2_ conditions experienced during early development had negative effects on the size and condition of juvenile *A. percula*. Interestingly, no interaction between juvenile CO_2_ and food level was observed, suggesting that negative trends reported in previous studies testing the effects of elevated CO_2_ with ample food^80^ could simply be magnified in low food conditions. Parental exposure to elevated CO_2_ induced enhanced linear growth and mitigated some of the negative effects of developing in elevated CO_2_ conditions, as has been observed previously^59^, however, this came at a cost of reduced physical condition. It seems that the importance of maintaining a particular length in a social hierarchy is likely to have produce this trade-off. Furthermore, the importance of the social system in this species perhaps explains why the size hierarchy was maintained across all combinations of juvenile stressors. Understanding the effects of acidification and food availability on marine animals within their natural social systems is an essential step to improve our ability to predict the effect of these environmental stressors on marine ecosystems. Ideally, future research should investigate the added impact of additional stressors such as ocean warming and sedimentation in conjunction with ocean acidification and food availability.

## Materials and Methods

### Study species

The orange clownfish, *Amphiprion percula*, is found on coral reefs of northern Australia, the Great Barrier Reef, and Melanesian Islands^81^. This species is known to display behavioural changes under elevated CO_2_ conditions^34,36,82,83^. Social groups of *A. percula* consist of a monogamous breeding pair and 0–8 non-breeding subordinates living in close association with a sea anemones^28,84^. The breeding pair are the largest individuals in the social group and any subordinates exhibit a size-based hierarchy, where each individual is approximately 10% smaller in length than the fish immediately above it in the size-based rank order^28^. Breeding pairs lay several clutches of >300 eggs throughout the summer breeding season on a hard substrate sheltered from flow^84^. The embryonic period lasts for 7–9 days^85^ and during this time the males tends to the eggs by fanning and removing unfertilized eggs and foreign material^86^. Upon hatching the larvae spend approximately 11 days in the pelagic larval stage before they are competent to settle on the reef  ^87^. All procedures were approved by the James Cook University Animal Ethics Committee (JCU Animal Ethics No. A2285) and all experiments were performed in accordance with the relevant guidelines and regulations.

### Parental experimental design

Breeding pairs of *A. percula* for this study were collected between 2011–2014 from the northern Great Barrier Reef and transported to the Marine and Aquaculture Research Facility at James Cook University, Townsville, Australia. Each pair was housed in an open-air 60 L aquaria with constant water flow and aeration. For this study, fourteen breeding pairs were randomly allocated into two CO_2_ treatment conditions (7 pairs in each treatment): a current-day control of 489 µatm CO_2_ and an elevated CO_2_ treatment of 1022 µatm CO_2_, consistent with projected future CO_2_ conditions for the ocean by the end of this century^88^. Parental CO_2_ treatments commenced four months prior to the beginning of the breeding period (September to December). Water temperature was increased at a rate of 0.25 °C per week from 26 °C (natural spring conditions) to 29 °C (natural summer conditions) over the same period to match the seasonal increase in water temperature prior to breeding.

Two 10,000 L recirculating seawater systems supplied seawater for the experiment. One system was maintained at ambient pCO_2_ (~489 µatm), similar to current-day conditions on coral reefs in summer^89^, while the second system was maintained at an elevated pCO_2_ to replicate end of century projections (~1022 µatm) (Table 1). The elevated CO_2_ treatment was achieved by dosing the water sump with CO_2_ to a predetermined pH setpoint following standard techniques^90^. The pH was regulated by a pH computer (Aquamedic AT-Contol) connected to a pH electrode and a solenoid valve, which maintained the desired pH by slowly dosing CO_2_ when pH deviated above the set point. The pH was cross-checked daily with a Mettler Toledo, SG9 pH meter and temperature was measured daily with a Comark-22 thermometer. Total alkalinity of each system was measured weekly by gran titration (Metrohm 888 titrando) to within 1% of certified reference material (Prof. Dickson, Scripps Oceanographic Institute). The pH_total_ of each weekly sample was measured by spectrophotometry (Shimadzu, UV mini 1240) and salinity measured with a conductivity probe (Hach HQ40d meter, IntelliCAL CDC401 probe). The pCO_2_ of each seawater samples was then calculated in CO2SYS^91^ from the measured values of total alkalinity, pH_total_, temperature and salinity and using the constants of Mehrbach, Cullberson, Hawley, & Pytkowicx^92^, refit by Dickson & Millero^93^ (Table 1).

**Table 1:** Mean (±SD) seawater chemistry parameters for *A. percula*, adults and juveniles, held under control and elevated CO_2_. Juvenile measurements were taken from the day the first clutch was laid until the last clutch reached 50 dph. Parental measurements were taken from the start of parental CO_2_ treatment until the last clutch was removed from parental care. Parents were maintained in treatments for 28 weeks before the first clutch was laid.

Treatment	Salinity (ppt)	Temperature (°C)	Total Alkalinity (µmol kg^−1^ SW)	pH (Total)	pCO_2_ (µatm)
Juvenile Ambient CO_2_	35.55 ± 0.70	28.38 ± 0.32	2121 ± 95	7.93 ± 0.03	489 ± 29
Juvenile Elevated CO_2_	35.83 ± 0.58	28.41 ± 0.40	2166 ± 104	7.66 ± 0.04	1022 ± 111
Parental Ambient CO_2_	35.05 ± 1.29	28.52 ± 0.77	2348 ± 279	7.97 ± 0.05	489 ± 37
Parental Elevated CO_2_	35.84 ± 1.02	28.35 ± 0.87	2415 ± 321	7.71 ± 0.05	1032 ± 95

Throughout the experiment, breeding pairs were fed to satiation daily with aquaculture pellets (Primo NRD size G12: protein 55%, lipids 9%, fibre 2%). Each pair was provided a terracotta half pot and tile below that acted as a spawning site for egg clutches. Tanks were visually inspected daily for new egg clutches and the progress of known clutches. Cleaning and siphoning of tanks was completed as required to remove any waste products and excess food.

### Larval rearing

**Figure 3:**
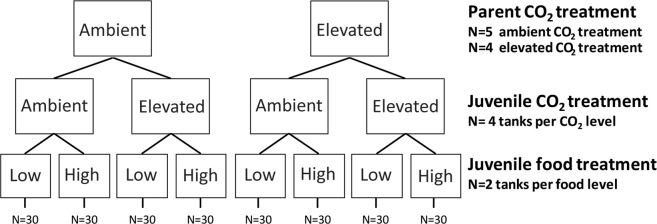
Experiment design tree. Parental pair were kept in treatment for four months leading up to the breeding season. Juveniles were moved into these treatments at 11 dph and reared until 50 dph.

Spawning occurred in 5 breeding pairs of *A. percula* from the current-day control group and 4 pairs from elevated CO_2_ treatment. A single egg clutch from each pair was used in the experiments. Egg clutches remained with the parents until the night of hatching to enable natural parental egg care to occur. Readiness to hatch was determined by visual inspection of eye development. On the night of hatching the half pot or tile with the eggs was removed from the parental tank and placed into a 100 L recirculating larval rearing tank with gentle aeration over the clutch. Once hatched, the flow of water into the 100 L tanks was alternated between open (~1 L/min) and closed (no water flow) depending on the time of day (0600–1800 closed, 1800–0600 open). This allowed for green-water larval rearing to be maintained during light hours and flushing of clean water during the night. Green-water rearing occurred for the first 4 days after which the tanks were switched to 24 hour continuous water flow (~1 L/min). All clutches were maintained in the same CO_2_ treatment water as their parents (i.e. 489 or 1022 µatm; Fig. 3). Larvae were maintained in the rearing tanks under their natal CO_2_ treatment for 11 days post-hatching (dph), representing the full pelagic development stage^94^. During the first 4 dph juveniles were fed once daily (at 0700) with rotifers at a density of 15–20 individuals ml^−1^ in their 100 L tank. Liquid algae (Nano 3600, Reed Mariculture) was mixed into the tank at 5 ml per 100 L to create a green water environment as per best practice^85,86^. From 4 dph onwards, juveniles were transitioned onto 12 hour *Artemia spp*. napuli (5–10 ml^−1^) along with rotifers at a concentration of 10 ml^−1^. From 6 dph rotifers were stopped and *Artemia spp*. napuli, 24 hours old, were provided (5–10 ml^−1^). Finally, at 8 dph juveniles were transitioned onto aquaculture feed (Primo Wean-L 0.3–5 mm), which was feed in excess once daily (~10 g per 100 L tank), and 24 h *Artemia spp*. napuli were reduced to 5 ml^−1^. This weaning protocol has been previously established as best practice^86^. At 11 dph fish were split between into the juvenile experimental treatments, as described below.

### Juvenile experimental design

Juvenile fish were reared in 35 L tanks inside a temperature controlled laboratory. Water temperature was maintained at 28.5 °C (±0.35 °C) throughout the experiment. At 11 dph juveniles from each clutch were split orthogonally between the two CO_2_ treatments (489 and 1022 µatm) and two food treatments (high and low) (Fig. 3). The food levels provided were relative to the average body weight of individuals and multiplied by the number of individual fish within a tank. The high food ration was 8% of average body weight (bw) and the low ration was 4% of average bw, fed once per day. These levels were determined from previous research showing that growth rate does not increase above 8% bw of food per day and 4% bw per day is sufficient to maintain basic growth^95^. An initial clutch was used to test the 8% ration and determine the mean growth rate throughout the 50 dph testing period. The weight of this pilot clutch was monitored weekly and used to calculate to amount of food to be given to each group of fish throughout the experiment. Specifically, food levels increased with each week in treatment (i.e. 18, 25, 32, 39 and 46 dph). For each of the four juvenile CO_2_ and food level treatment combinations, there were two replicate tanks per clutch with 15 individuals in each tank (Fig. 3). All tanks were checked daily and any mortalities were recorded. Survival was >75% in all groups regardless of treatment combination. Juveniles were reared under these treatments until 50 dph at which point they were euthanized with clove oil solution (1 ml clove oil/200 ml seawater). All individuals were photographed with a Cannon G9X camera (macro setting in the presence of a 100 mm scale bar) and measured for wet weight (to nearest 0.0001 g). Photos were analysed to determine the standard length of each fish (to nearest 0.01 mm) using ImageJ software. The standard length and wet weight was then used to calculate Fulton’s K index of condition (*K* = *100(Weight/Length*^3^), where fish that are heavier for a given length have a higher condition value.

Juvenile *A. percula* were stocked at 15 individuals per tank to account for attrition, any mortalities, and ensure a viable number of individuals at the endpoint of the experiment. At 50 dph survival was >75% in all tanks with 11 to 15 individuals. The goal of the present study was to test the effects of parental CO_2_ exposure, juvenile CO_2_ exposure and food ration on the growth and condition of juveniles, as well as any effects to the size hierarchy. Since *A. percula* have not been found in colonies of more than 10 individuals^28,83^, only the fish in ranks 1 to 10 (i.e. the 10 largest individuals) in each tank were included in subsequent analyses.

### Data analysis

The length, weight and Fulton’s K of individuals was compared among treatments using linear mixed effect models (LME) fitted with residual maximum likelihood (REML). An LME was used to determine the relationship between each of the response variables (i.e. length, weight or Fulton’s K) and the independent variables (parental CO_2_ treatment, juvenile CO_2_ treatment, food level and hierarchical rank within tank). A full factorial model was used to test all possible interactions. Parent ID and rearing tank ID were used as random factors in all models. The length of the largest individual from each tank (rank 1 individuals) was also compared among treatments using LME, with length as the response variable and parental CO_2_ treatment, juvenile CO_2_ treatment, food level as the independent variables, in a full factorial model. A generalised linear mixed model (GLMM) was used to analyse the body size ratios between ranks. This GLMM tested for any relationship between the response variable (body size ratio frequency) and the independent variables (parental CO_2_ treatment, juvenile CO_2_ treatment, food level) using a fully factorial design. Parent ID and rearing tank ID were used as random factors in all models. Backwards stepwise removal of non-significant interactions was conducted (AIC comparisons) on all models to identify any possible significant interaction not seen in the full factorial model, however, there were no significant differences found with this method, therefore the full models were maintained. Significant interactions (p < 0.05) identified in mixed models were further investigated with a Tukey’s post-hoc test to explore the differences. Bonferroni correction was applied to reduce the potential of false detection. The statistical analyses were conducted in IMB SPSS 25.

## Supplementary information

Supplementary Information

## Data Availability

Data from this paper is available on The Tropical Data Hub 10.25903/5df182c2f5350.
