# Supplementary material for: Elevated CO2 and food ration affect growth but not the size-based hierarchy of a reef fish
Source: Sci Rep. 2019 Dec 23;9:19706. doi: 10.1038/s41598-019-56002-z (PMC6928027; doi:10.1038/s41598-019-56002-z)

***Supplementary materials for:***

# “Elevated CO_2_ and food ration affect growth but not the size-based hierarchy of a reef fish”

Shannon J. McMahon^1^, Philip L. Munday^1^, Marian Y.L. Wong^2^, Jennifer M. Donelson^1^

^1^ARC Centre of Excellence for Coral Reef Studies, James Cook University, Townsville, QLD 4811, Australia

^2^Centre for Sustainable Ecosystems Solutions, School of Biological Sciences, University of Wollongong, Wollongong, NSW 2522, Australia

*Corresponding author:

Shannon McMahon

ARC Centre of Excellence for Coral Reef Studies, James Cook University, Townsville, QLD 4811, Australia

Email address: [Shannon.mcmahon@jcu.edu.au](mailto:Shannon.mcmahon@jcu.edu.au)


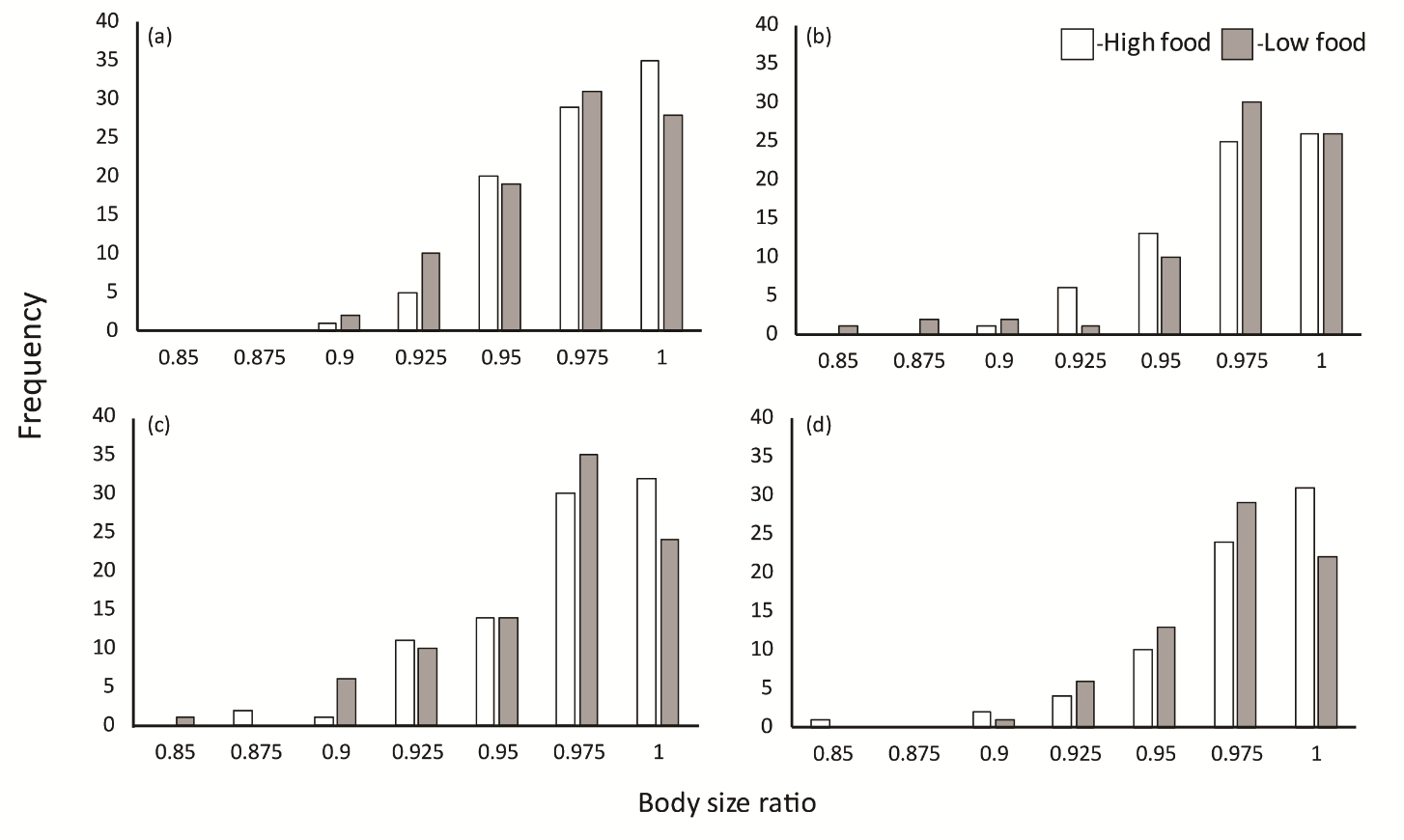


**Figure S1**. Frequency of body ratio between ranked individuals of juvenile *Amphiprion percula* at 50 dph, where (a) parents and juveniles were at ambient CO_2_ (489 µatm), (b) parents were at elevated CO_2_ (1032 µatm) and juveniles were at ambient CO_2_ (489 µatm), (c) parents were at ambient CO_2_ (489 µatm) and juveniles were at elevated CO_2_ (1022 µatm), and (d) both parents and juveniles were at elevated CO_2_ (1032/1022 µatm). Grey bars represent juveniles reared under the high food treatment, while white bars represent juveniles reared under the low food treatment.

**Table S1**. Linear mixed effects models on the length of juvenile A. percula. Juveniles were reared in either high or low food treatment cross factored with ambient (489 µatm) or elevated CO_2_ (1022 µatm). Juveniles were from parents exposed to either control (489 µatm) or elevated CO_2_ (1032 µatm).


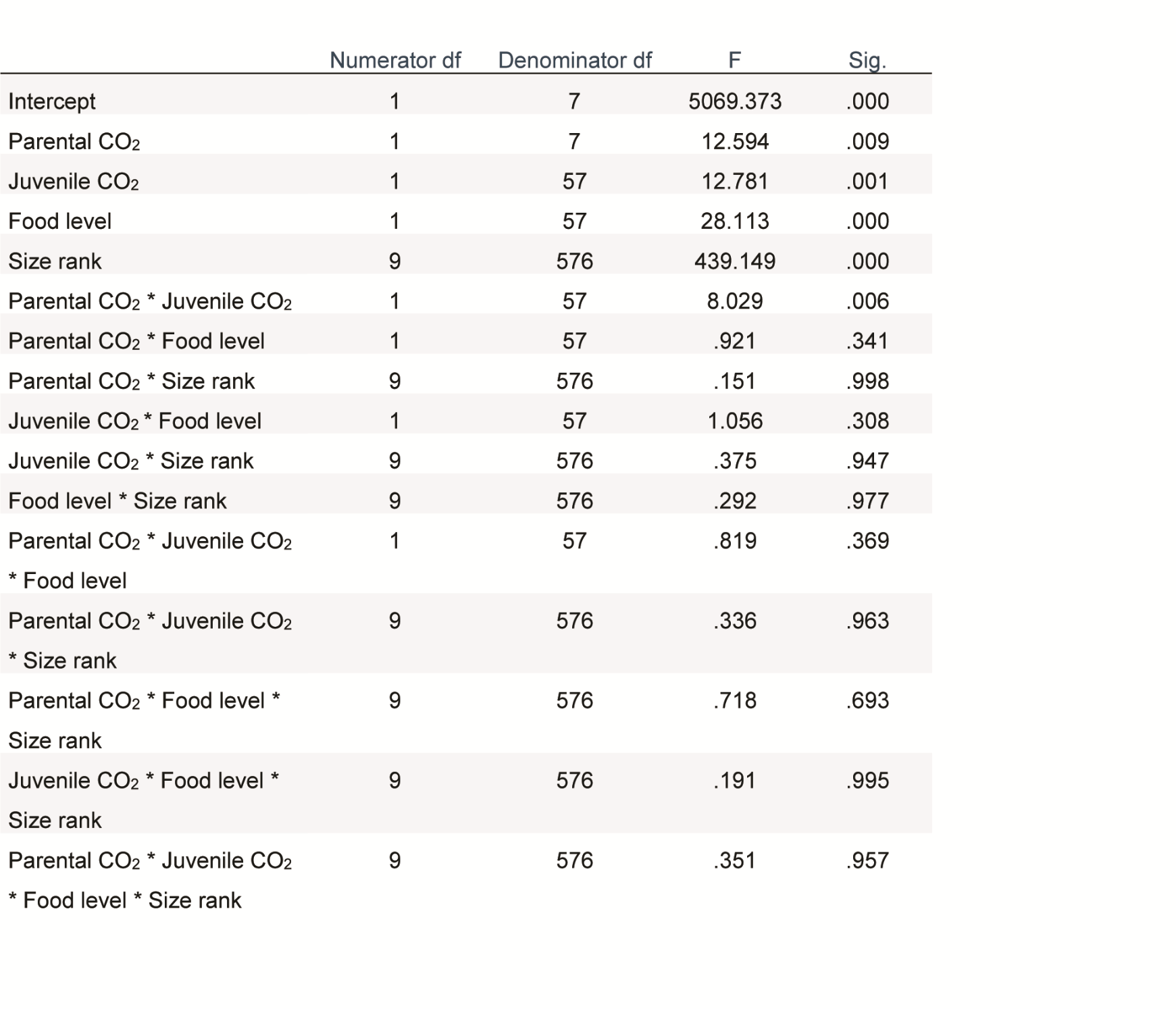


**Table S2**. Linear mixed effects models on the weight of juvenile *A. percula*. Juveniles were reared in either high or low food treatment cross factored with *ambient* (489 µatm) or elevated CO_2_ (1022 µatm). Juveniles were from parents exposed to either control (489 µatm) or elevated CO_2_ (1032 µatm).
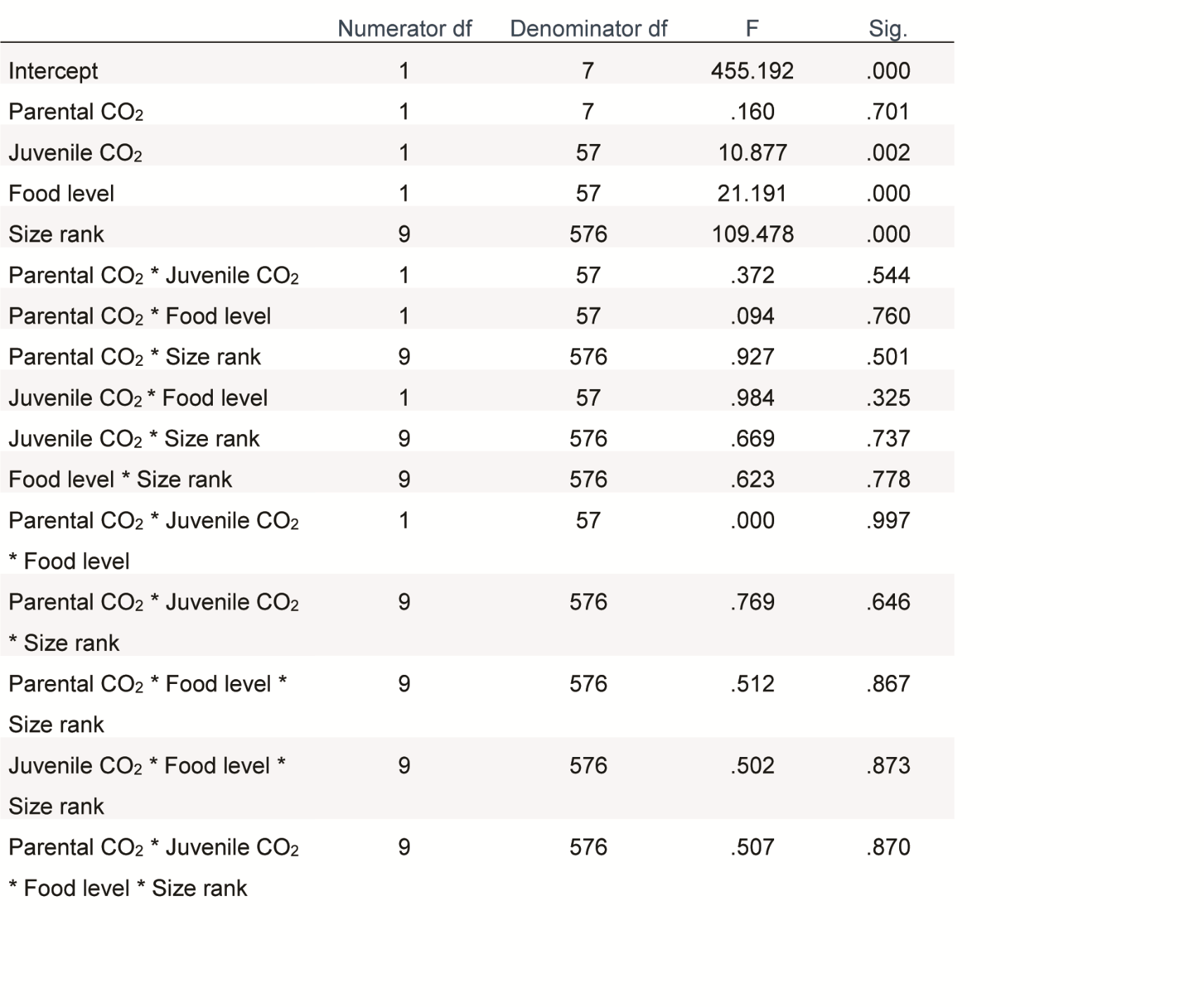


**Table S3.** Linear mixed effects models on the Fulton’s K of juvenile *A. percula*. Juveniles were reared in either high or low food treatment cross factored with *ambient* (489 µatm) or elevated CO_2_ (1022 µatm). Juveniles were from parents exposed to either control (489 µatm) or elevated CO_2_ (1032 µatm).
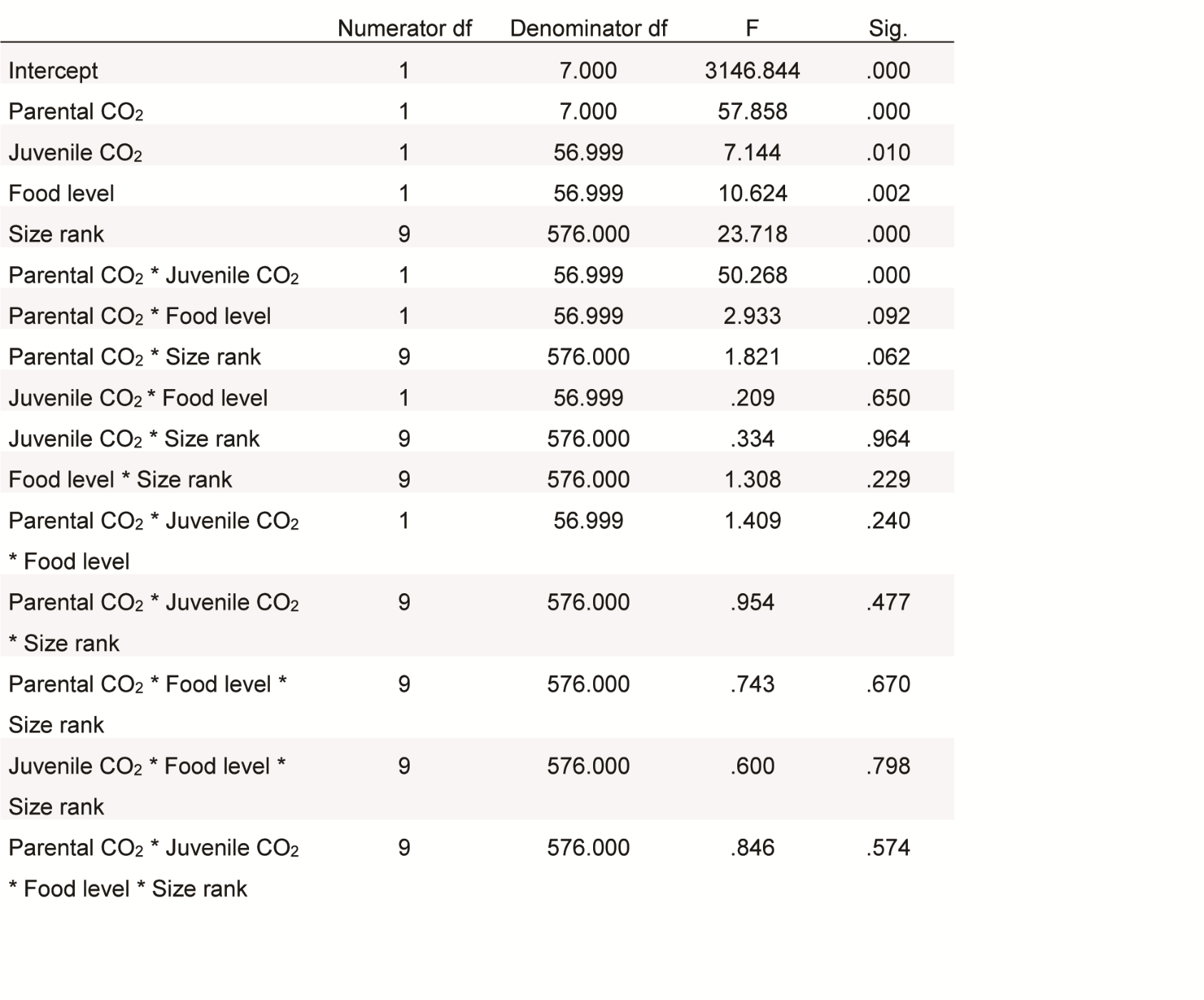


**Table S4.** Generalised linear mixed effects models on the body size ratio frequency between ranks of *juvenile A. percula*. Juveniles were reared in either high or low food treatment cross factored with *ambient* (489 µatm) or elevated CO_2_ (1022 µatm). Juveniles were from parents exposed to either control (489 µatm) or elevated CO_2_ (1032 µatm).
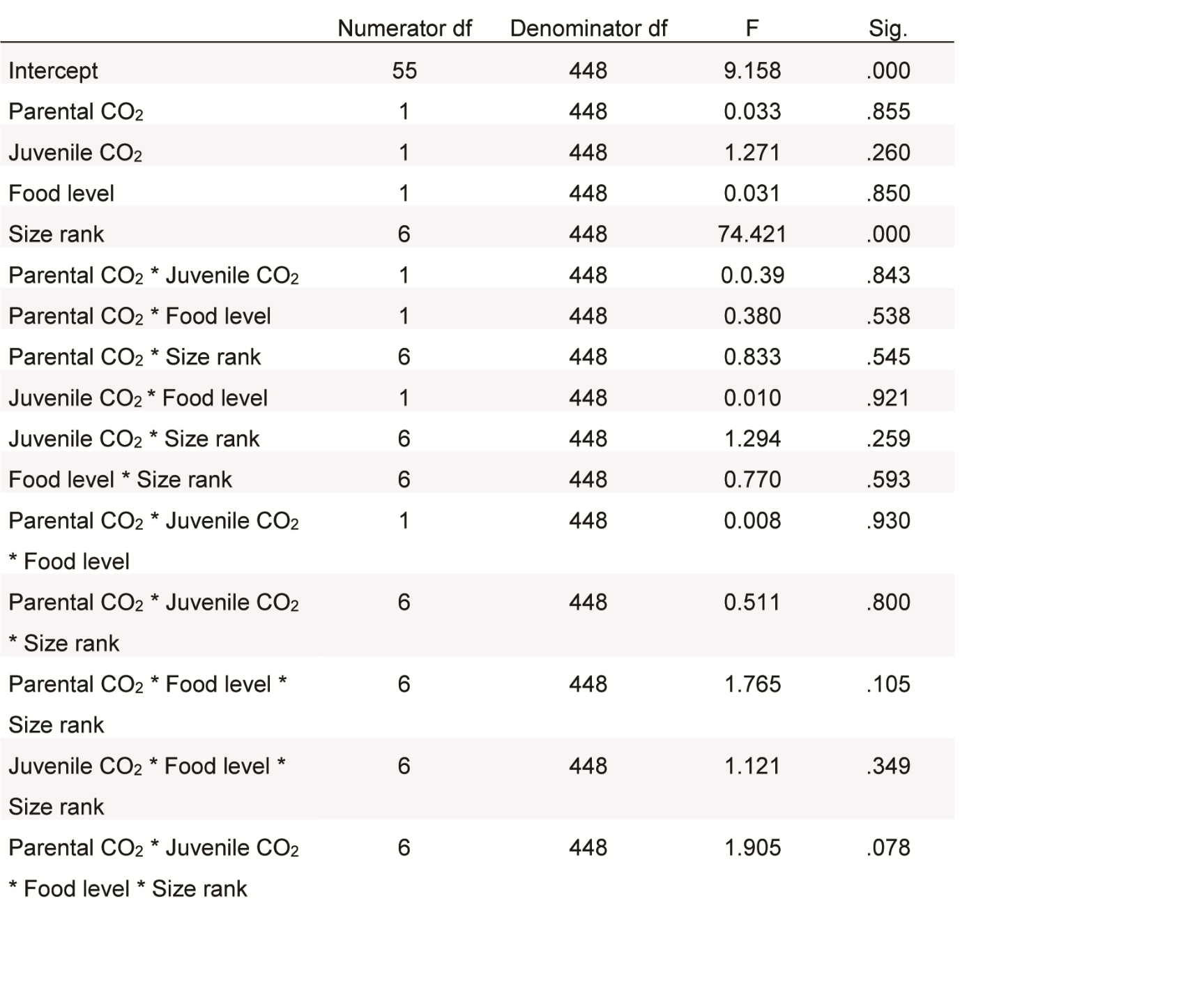


**Table S5.** Linear mixed effects models on the length of rank 1 individuals. Juvenile *A. percula* were reared in either high or low food treatment cross factored with ambient (489 µatm) or elevated CO_2_ (1022 µatm). Juveniles were from parents exposed to either control (489 µatm) or elevated CO_2_ (1032 µatm).
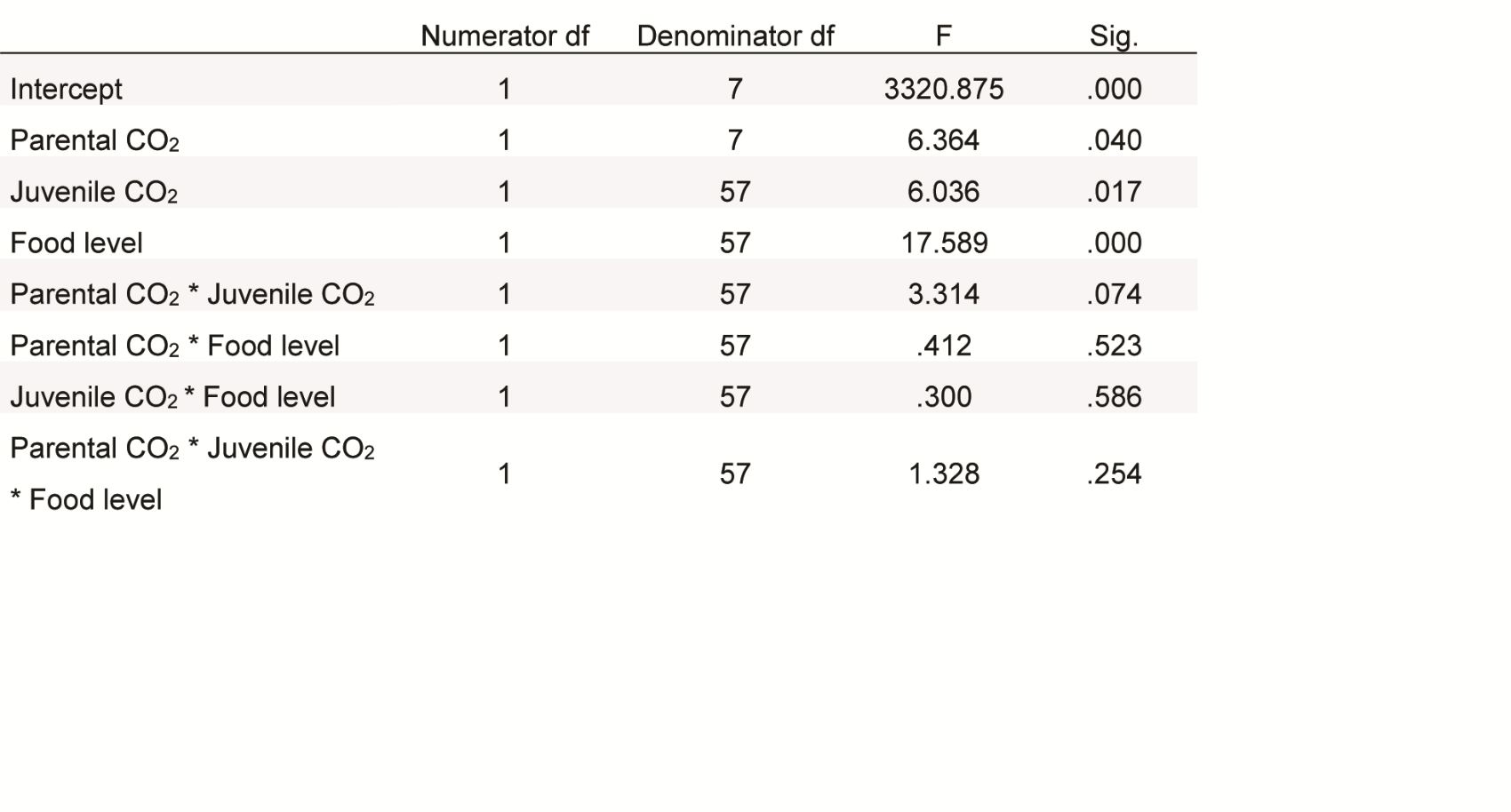


**Table S6**. Tukey’s post-hoc comparison on length of juvenile A. percula, Contrasts were conducted on the parental (P) and Juvenile (J) CO_2_ levels, ambient (489 µatm) and elevated (1022 µatm for juveniles, 1032 µatm for parents). Using a Bonferroni correction significance was adjusted to p=0.0083.


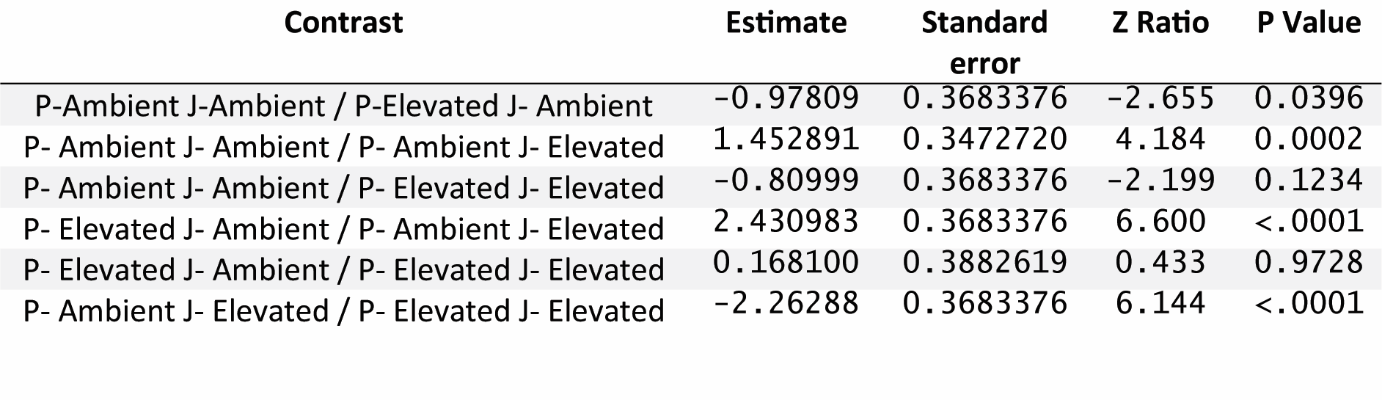


**Table S7.** Tukey’s post*-*hoc comparison on Fulton’s K *of juvenile A. percula, Contrasts were conducted on t*he parental (P) and Juvenile (J) CO_2_ levels*, a*mbient (489 µatm) and elevated (1022 µatm for juveniles, 1032 µatm for parents). Using a Bonferroni correction significance was adjusted to p=0.0083*.*
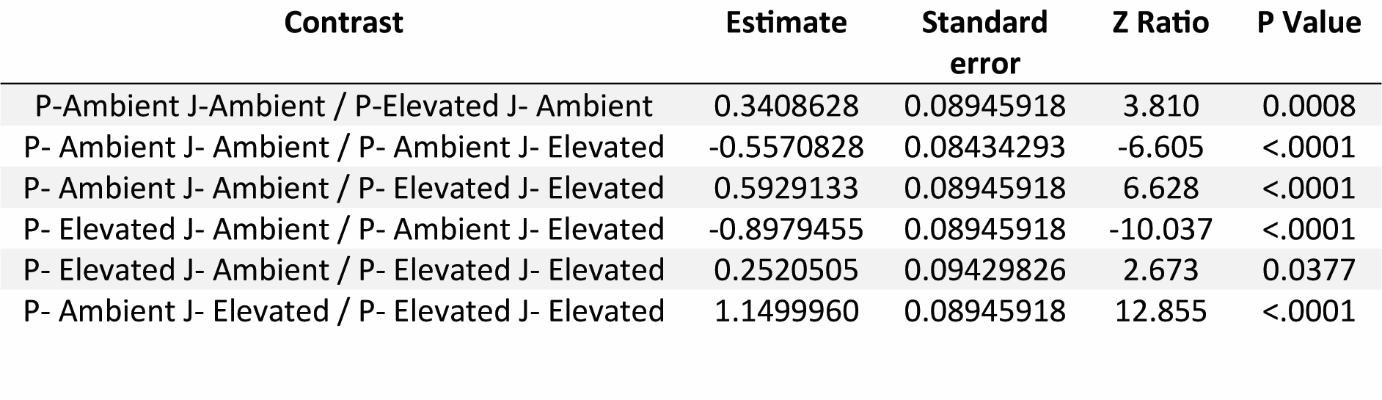

Supplement: Supplementary file 1 — Supplementary Information [file 41598_2019_56002_MOESM1_ESM.docx]
